# 2-[(*E*)-(4-Fluoro­benz­yl)imino­meth­yl]-6-meth­oxy­phenol

**DOI:** 10.1107/S1600536812027419

**Published:** 2012-07-10

**Authors:** Hong Yu, Yue-Bao Jin, Yong-Kang Chang, Ke-Wei Lei

**Affiliations:** a State Key Lab. Base of Novel Functional Materials and Preparation Science, Institute of Solid Materials Chemistry, Faculty of Materials Science and Chemical Engineering, Ningbo University, Ningbo 315211, People’s Republic of China

## Abstract

In the title Schiff base, C_15_H_14_FNO_2_, the dihedral angle between the benzene rings is 53.32 (8)°. In the crystal, mol­ecules related by a twofold rotation axis are linked by pairs of C—H⋯O hydrogen bonds into dimers with *R*_2_^2^(18) ring motifs. An intra­molecular O—H⋯N hydrogen bond is also observed.

## Related literature

For general background to Schiff base complexs which show photochromism and thermochromism in the solid state, see: Cohen *et al.* (1964 ▶). For a related structure, see: Li *et al.* (2007 ▶).
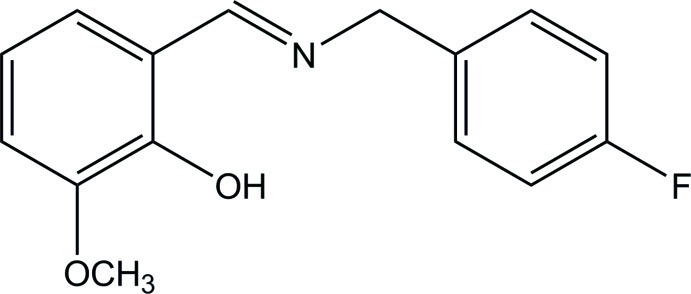


## Experimental

### 

#### Crystal data

C_15_H_14_FNO_2_
*M* *_r_* = 259.27
Monoclinic, 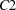

*a* = 20.5577 (15) Å
*b* = 5.5281 (3) Å
*c* = 13.1315 (9) Å
β = 118.477 (9)°
*V* = 1311.77 (19) Å^3^
*Z* = 4
Mo *K*α radiation
μ = 0.10 mm^−1^
*T* = 293 K
0.43 × 0.25 × 0.16 mm

#### Data collection

Rigaku R-AXIS RAPID diffractometer
Absorption correction: multi-scan (*ABSCOR*; Higashi, 1995 ▶) *T*_min_ = 0.971, *T*_max_ = 0.985
5883 measured reflections
1902 independent reflections
1525 reflections with *I* > 2σ(*I*)
*R*_int_ = 0.021

#### Refinement

*R*[*F* ^2^ > 2σ(*F* ^2^)] = 0.042
*wR*(*F* ^2^) = 0.125
*S* = 1.17
1902 reflections
175 parameters
2 restraints
H atoms treated by a mixture of independent and constrained refinement
Δρ_max_ = 0.12 e Å^−3^
Δρ_min_ = −0.15 e Å^−3^

### 

Data collection: *RAPID-AUTO* (Rigaku, 1998 ▶); cell refinement: *RAPID-AUTO*; data reduction: *CrystalStructure* (Rigaku/MSC, 2004 ▶); program(s) used to solve structure: *SHELXS97* (Sheldrick, 2008 ▶); program(s) used to refine structure: *SHELXL97* (Sheldrick, 2008 ▶); molecular graphics: *SHELXTL* (Sheldrick, 2008 ▶); software used to prepare material for publication: *SHELXL97*.

## Supplementary Material

Crystal structure: contains datablock(s) global, I. DOI: 10.1107/S1600536812027419/is5154sup1.cif

Structure factors: contains datablock(s) I. DOI: 10.1107/S1600536812027419/is5154Isup2.hkl

Supplementary material file. DOI: 10.1107/S1600536812027419/is5154Isup3.cml

Additional supplementary materials:  crystallographic information; 3D view; checkCIF report

## Figures and Tables

**Table 1:** Hydrogen-bond geometry (Å, °)

*D*—H⋯*A*	*D*—H	H⋯*A*	*D*⋯*A*	*D*—H⋯*A*
O1—H1⋯N1	0.85 (4)	1.81 (4)	2.597 (4)	154 (3)
C15—H15*A*⋯O1^i^	0.93	2.53	3.442 (4)	166

Symmetry code: (i) 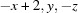.

**Fig. 1.**
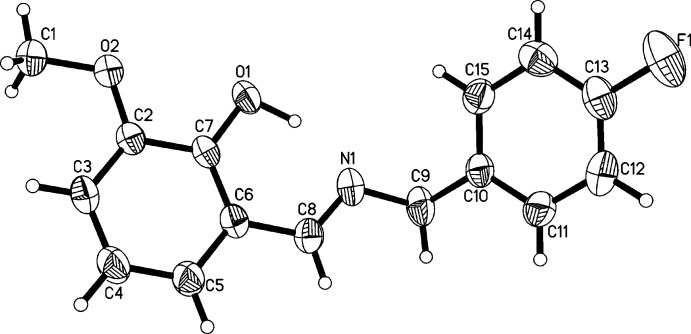
The molecular structure of the title compound, showing 30% probability displacement ellipsoids and the atom-numbering scheme.

## supplementary crystallographic information

### Comment

Schiff bases ligands have been used with remarkable success in inorganic and organometallic chemistry over past decades. Some of the reasons are that Schiff bases have good coordination ability with transition metals and that Schiff bases complexs show photochromism and thermochromism in the solid state by proton transfer from the hydroxyl O atom to the imine N atom (Cohen *et al.*, 1964). Here, we report the structure of a new Schiff base. A similar Schiff base molecule has been reported by Li *et al.* (2007). The molecular structure is illustrated in Fig. 1. The dihedral angle between two benzenes rings is 53.32 (8)°. There are an intramolecular O1—H1···N1 hydrogen bond and an intermolecular C15—H15A···O1 hydrogen bond (Table 1).

### Experimental

2-Hydroxy-3-methoxybenzaldehyde (20 mmol,3.0 g) and (4-fluorophenyl)methanamine (20 mmol,2.5 g) dissolved in ethanol respectively. Then put them together and the solution was refluxed for 1 h. After evaporation, a crude product was recrystallized twice from ethanol to give a pure yellow product (yield 88.3%). Calcd. for C_15_H_14_FNO_2_: C 69.49, H 5.44, O 12.34, N 5.40%; Found: C 69.71, H 5.46, O 12.35, N 5.41%

### Refinement

O-bound H atom was located in a difference Fourier map and its position was refined with a restraint of O—H = 0.82 (2) Å and with *U*_iso_(H) = 1.5*U*_eq_(O). Other H atoms were placed in geometrically idealized positions (C—H = 0.93–0.97 Å) and constrained to ride on their parent atoms with *U*_iso_(H) = 1.2*U*_eq_(C) or 1.5*U*_eq_(C_methyl_) .

### Crystal data

**Table:**

C_15_H_14_FNO_2_	*F*(000) = 544
*M**_r_* = 259.27	*D*_x_ = 1.313 Mg m^−^^3^
Monoclinic, *C*2	Mo *K*α radiation, λ = 0.71073 Å
Hall symbol: C 2y	Cell parameters from 3354 reflections
*a* = 20.5577 (15) Å	θ = 1.0–28.9°
*b* = 5.5281 (3) Å	µ = 0.10 mm^−^^1^
*c* = 13.1315 (9) Å	*T* = 293 K
β = 118.477 (9)°	Block, yellow
*V* = 1311.77 (19) Å^3^	0.43 × 0.25 × 0.16 mm
*Z* = 4	

### Data collection

**Table:**

Rigaku R-AXIS RAPID diffractometer	1902 independent reflections
Radiation source: fine-focus sealed tube	1525 reflections with *I* > 2σ(*I*)
Graphite monochromator	*R*_int_ = 0.021
ω scans	θ_max_ = 28.9°, θ_min_ = 3.2°
Absorption correction: multi-scan (*ABSCOR*; Higashi, 1995)	*h* = −25→27
*T*_min_ = 0.971, *T*_max_ = 0.985	*k* = −6→7
5883 measured reflections	*l* = −16→17

### Refinement

**Table:**

Refinement on *F*^2^	Primary atom site location: structure-invariant direct methods
Least-squares matrix: full	Secondary atom site location: difference Fourier map
*R*[*F*^2^ > 2σ(*F*^2^)] = 0.042	Hydrogen site location: inferred from neighbouring sites
*wR*(*F*^2^) = 0.125	H atoms treated by a mixture of independent and constrained refinement
*S* = 1.17	*w* = 1/[σ^2^(*F*_o_^2^) + (0.049*P*)^2^ + 0.4613*P*] where *P* = (*F*_o_^2^ + 2*F*_c_^2^)/3
1902 reflections	(Δ/σ)_max_ = 0.001
175 parameters	Δρ_max_ = 0.12 e Å^−^^3^
2 restraints	Δρ_min_ = −0.15 e Å^−^^3^

### Special details

**Table:**

Geometry. All e.s.d.'s (except the e.s.d. in the dihedral angle between two l.s. planes) are estimated using the full covariance matrix. The cell e.s.d.'s are taken into account individually in the estimation of e.s.d.'s in distances, angles and torsion angles; correlations between e.s.d.'s in cell parameters are only used when they are defined by crystal symmetry. An approximate (isotropic) treatment of cell e.s.d.'s is used for estimating e.s.d.'s involving l.s. planes.
Refinement. Refinement of *F*^2^ against ALL reflections. The weighted *R*-factor *wR* and goodness of fit *S* are based on *F*^2^, conventional *R*-factors *R* are based on *F*, with *F* set to zero for negative *F*^2^. The threshold expression of *F*^2^ > σ(*F*^2^) is used only for calculating *R*-factors(gt) *etc*. and is not relevant to the choice of reflections for refinement. *R*-factors based on *F*^2^ are statistically about twice as large as those based on *F*, and *R*- factors based on ALL data will be even larger.

### Fractional atomic coordinates and isotropic or equivalent isotropic displacement parameters (Å^2^)

**Table:**

	*x*	*y*	*z*	*U*_iso_*/*U*_eq_	
F1	0.61623 (12)	0.1593 (6)	−0.32616 (19)	0.1177 (10)	
O1	1.02596 (11)	0.0073 (5)	0.12995 (15)	0.0681 (6)	
H1	0.9934 (18)	0.112 (6)	0.091 (3)	0.102*	
O2	1.12802 (12)	−0.2598 (5)	0.29122 (16)	0.0744 (6)	
N1	0.93995 (12)	0.3831 (5)	0.07357 (19)	0.0660 (7)	
C1	1.18810 (18)	−0.3918 (8)	0.3777 (3)	0.0859 (11)	
H1C	1.2044	−0.5110	0.3417	0.129*	
H1D	1.1725	−0.4706	0.4276	0.129*	
H1E	1.2281	−0.2832	0.4225	0.129*	
C2	1.09825 (14)	−0.0822 (6)	0.3284 (2)	0.0560 (6)	
C3	1.11648 (16)	−0.0370 (6)	0.4429 (2)	0.0656 (8)	
H3A	1.1504	−0.1362	0.5013	0.079*	
C4	1.08473 (18)	0.1536 (8)	0.4710 (2)	0.0787 (10)	
H4A	1.0971	0.1801	0.5480	0.094*	
C5	1.03524 (16)	0.3046 (7)	0.3869 (2)	0.0712 (9)	
H5A	1.0154	0.4355	0.4072	0.085*	
C6	1.01464 (13)	0.2608 (6)	0.2699 (2)	0.0560 (6)	
C7	1.04497 (13)	0.0636 (5)	0.24101 (19)	0.0523 (6)	
C8	0.96436 (14)	0.4252 (6)	0.1806 (2)	0.0622 (7)	
H8A	0.9496	0.5659	0.2027	0.075*	
C9	0.89012 (17)	0.5601 (7)	−0.0103 (3)	0.0749 (9)	
H9A	0.9126	0.6235	−0.0550	0.090*	
H9B	0.8825	0.6939	0.0307	0.090*	
C10	0.81621 (15)	0.4466 (6)	−0.0912 (2)	0.0582 (7)	
C11	0.75116 (18)	0.5559 (7)	−0.1113 (3)	0.0716 (8)	
H11A	0.7524	0.6962	−0.0713	0.086*	
C12	0.68320 (17)	0.4581 (8)	−0.1912 (3)	0.0822 (11)	
H12A	0.6392	0.5332	−0.2055	0.099*	
C13	0.68242 (18)	0.2542 (8)	−0.2471 (3)	0.0759 (9)	
C14	0.7451 (2)	0.1359 (7)	−0.2279 (3)	0.0770 (9)	
H14A	0.7427	−0.0069	−0.2669	0.092*	
C15	0.81264 (17)	0.2330 (7)	−0.1487 (2)	0.0698 (8)	
H15A	0.8561	0.1536	−0.1339	0.084*	

### Atomic displacement parameters (Å^2^)

**Table:**

	*U*^11^	*U*^22^	*U*^33^	*U*^12^	*U*^13^	*U*^23^
F1	0.0827 (12)	0.156 (3)	0.0899 (13)	−0.0452 (16)	0.0215 (11)	0.0028 (16)
O1	0.0649 (11)	0.0837 (15)	0.0414 (9)	0.0055 (11)	0.0138 (8)	−0.0017 (10)
O2	0.0735 (12)	0.0749 (14)	0.0549 (10)	0.0165 (12)	0.0144 (9)	−0.0033 (10)
N1	0.0544 (12)	0.0786 (17)	0.0537 (12)	0.0012 (12)	0.0166 (10)	0.0137 (12)
C1	0.0739 (19)	0.088 (3)	0.0702 (18)	0.022 (2)	0.0138 (15)	−0.0022 (19)
C2	0.0492 (12)	0.0625 (16)	0.0497 (12)	−0.0032 (12)	0.0182 (10)	−0.0007 (12)
C3	0.0625 (15)	0.080 (2)	0.0453 (12)	0.0042 (16)	0.0181 (11)	0.0115 (14)
C4	0.083 (2)	0.107 (3)	0.0460 (13)	0.017 (2)	0.0306 (14)	0.0048 (17)
C5	0.0669 (16)	0.092 (2)	0.0543 (14)	0.0140 (17)	0.0285 (13)	0.0026 (16)
C6	0.0465 (12)	0.0715 (18)	0.0467 (11)	−0.0016 (13)	0.0196 (10)	0.0037 (13)
C7	0.0447 (11)	0.0636 (16)	0.0421 (11)	−0.0078 (11)	0.0154 (9)	0.0000 (11)
C8	0.0511 (13)	0.0699 (18)	0.0619 (14)	0.0007 (14)	0.0240 (11)	0.0071 (14)
C9	0.0664 (16)	0.076 (2)	0.0617 (16)	−0.0017 (16)	0.0134 (13)	0.0197 (16)
C10	0.0582 (14)	0.0603 (17)	0.0491 (12)	0.0050 (13)	0.0199 (11)	0.0135 (12)
C11	0.0760 (18)	0.0661 (18)	0.0674 (17)	0.0123 (16)	0.0300 (15)	0.0097 (15)
C12	0.0578 (16)	0.100 (3)	0.085 (2)	0.0137 (19)	0.0313 (15)	0.023 (2)
C13	0.0671 (18)	0.092 (3)	0.0594 (15)	−0.014 (2)	0.0224 (14)	0.0133 (19)
C14	0.093 (2)	0.071 (2)	0.0661 (16)	−0.012 (2)	0.0372 (16)	−0.0023 (17)
C15	0.0680 (17)	0.0727 (19)	0.0660 (16)	0.0116 (17)	0.0297 (14)	0.0089 (16)

### Geometric parameters (Å, º)

**Table:**

F1—C13	1.363 (4)	C5—H5A	0.9300
O1—C7	1.354 (3)	C6—C7	1.396 (4)
O1—H1	0.846 (19)	C6—C8	1.454 (4)
O2—C2	1.364 (4)	C8—H8A	0.9300
O2—C1	1.418 (4)	C9—C10	1.514 (4)
N1—C8	1.269 (4)	C9—H9A	0.9700
N1—C9	1.464 (4)	C9—H9B	0.9700
C1—H1C	0.9600	C10—C11	1.374 (4)
C1—H1D	0.9600	C10—C15	1.384 (5)
C1—H1E	0.9600	C11—C12	1.396 (5)
C2—C3	1.388 (4)	C11—H11A	0.9300
C2—C7	1.403 (4)	C12—C13	1.341 (6)
C3—C4	1.379 (5)	C12—H12A	0.9300
C3—H3A	0.9300	C13—C14	1.356 (5)
C4—C5	1.371 (5)	C14—C15	1.386 (4)
C4—H4A	0.9300	C14—H14A	0.9300
C5—C6	1.406 (4)	C15—H15A	0.9300
			
C7—O1—H1	103 (3)	N1—C8—C6	122.2 (3)
C2—O2—C1	116.9 (2)	N1—C8—H8A	118.9
C8—N1—C9	118.4 (3)	C6—C8—H8A	118.9
O2—C1—H1C	109.5	N1—C9—C10	111.1 (3)
O2—C1—H1D	109.5	N1—C9—H9A	109.4
H1C—C1—H1D	109.5	C10—C9—H9A	109.4
O2—C1—H1E	109.5	N1—C9—H9B	109.4
H1C—C1—H1E	109.5	C10—C9—H9B	109.4
H1D—C1—H1E	109.5	H9A—C9—H9B	108.0
O2—C2—C3	125.6 (3)	C11—C10—C15	118.5 (3)
O2—C2—C7	115.4 (2)	C11—C10—C9	120.8 (3)
C3—C2—C7	119.0 (3)	C15—C10—C9	120.7 (3)
C4—C3—C2	120.6 (3)	C10—C11—C12	120.5 (3)
C4—C3—H3A	119.7	C10—C11—H11A	119.7
C2—C3—H3A	119.7	C12—C11—H11A	119.7
C5—C4—C3	121.0 (3)	C13—C12—C11	118.9 (3)
C5—C4—H4A	119.5	C13—C12—H12A	120.5
C3—C4—H4A	119.5	C11—C12—H12A	120.5
C4—C5—C6	119.7 (3)	C12—C13—C14	122.8 (3)
C4—C5—H5A	120.1	C12—C13—F1	119.2 (4)
C6—C5—H5A	120.1	C14—C13—F1	118.1 (4)
C7—C6—C5	119.4 (3)	C13—C14—C15	118.4 (3)
C7—C6—C8	120.5 (2)	C13—C14—H14A	120.8
C5—C6—C8	120.1 (3)	C15—C14—H14A	120.8
O1—C7—C6	122.4 (2)	C10—C15—C14	120.9 (3)
O1—C7—C2	117.4 (2)	C10—C15—H15A	119.6
C6—C7—C2	120.2 (2)	C14—C15—H15A	119.6

### Hydrogen-bond geometry (Å, º)

**Table:**

*D*—H···*A*	*D*—H	H···*A*	*D*···*A*	*D*—H···*A*
O1—H1···N1	0.85 (4)	1.81 (4)	2.597 (4)	154 (3)
C15—H15*A*···O1^i^	0.93	2.53	3.442 (4)	166

Symmetry code: (i) −*x*+2, *y*, −*z*.
